# The Effect of Trait Self-Awareness, Self-Reflection, and Perceptions of Choice Meaningfulness on Indicators of Social Identity within a Decision-Making Context

**DOI:** 10.3389/fpsyg.2017.02034

**Published:** 2017-11-30

**Authors:** Noam Dishon, Julian A. Oldmeadow, Christine Critchley, Jordy Kaufman

**Affiliations:** ^1^Department of Psychological Sciences, Faculty of Health, Arts and Design, Swinburne University of Technology, Melbourne, VIC, Australia; ^2^Department of Statistics, Data Science and Epidemiology, Faculty of Health, Arts and Design, Swinburne University of Technology, Melbourne, VIC, Australia

**Keywords:** self-reflective reasoning, self-awareness, subjective meaningfulness, self-concept, minimal group paradigm, in-group identification

## Abstract

Theorists operating from within a narrative identity framework have suggested that self-reflective reasoning plays a central role in the development of the self. Typically, however, narrative identity researchers have investigated this relationship using correlational rather than experimental methods. In the present study, leveraging on a classic research paradigm from within the social identity literature we developed an experiment to test the extent to which self-reflection might have a causal impact on the self-concept within a decision-making context. In a minimal group paradigm participants were prompted to reflect on their painting choices either before or after allocating points to in-group∖ out-group members. As anticipated, self-reflection augmented social identification, but only when participants felt their choices were personally meaningful. Participants who reasoned about their choices and felt they were subjectively meaningful showed stronger similarity and liking for in-group members compared to those who did not reflect on their choices or found them to be subjectively meaningless. Hence, reflecting on and finding meaning in one’s choices may be an important step in linking behavior with in-group identification and thus the self-concept in turn. The absence of any effects on in-group favoritism (a third indicator of social identification measured) as well as implications of the study’s findings for self-perception, cognitive dissonance and social identity processes are also discussed.

## Introduction

Psychological scientists have approached the issue of self and identity from a range of different positions. For example, some social and cultural psychologists have investigated self and identity using a social identity theory framework whereas other personality and developmental psychologists have pursued an approach informed by narrative identity theory (see, Tajfel and Turner, 1986; McAdams, 2001; Pasupathi et al., 2007; Miramontez et al., 2008). In the present paper, we synthesize aspects of both identity projects by utilizing an experimental paradigm associated with social identity theory (i.e., the minimal group paradigm), to investigate whether self-reflective reasoning, a cognitive process theorized to be central to narrative identity development, can have a causal effect on the self and identity. We also explore if such an effect could be impacted by the level of meaningfulness one associates with their self-reflective reasoning and modulated by individual differences in trait self-awareness.

### Identity from a Narrative Identity Framework

McAdams (1985, 2001) model of narrative identity postulates that our sense of identity is inextricably linked with the creation of a life story. According to this model, self-narratives have two primary functions. They facilitate our sense of self-continuity across time and they help us give context and meaning to the events of our lives so that we can make sense of who we are (McAdams and McLean, 2013). Self-narratives, as McAdams and McLean (2013), state facilitate meaning making because they allow the narrator to draw “…a semantic conclusion about the self from the episodic information that the story conveys” (pp. 236). Within the narrative identity literature the process of self-reflection coupled with the extraction of self-relevant meaning is referred to as autobiographical reasoning and it is theorized to be an essential cognitive process in narrative identity development and construction (Singer et al., 2013). However, as Adler et al. (2016) note, within the narrative identity literature investigators have typically employed correlational research designs thereby rendering it difficult to draw causal conclusions. Adler et al. (2016) develop this idea further stating that given this paucity of experimental work “increasing methodological sophistication and variety in the study of narrative identity with an eye toward drawing causal inferences is vital” (pp. 29).

### Self-Reflection, Meaning and the Self

Although research from within the narrative identity literature demonstrating a causal link between self-reflection and identity development remains scarce, several other lines of converging research also suggest that self-reflection should play an important role in self-concept development. For example within the clinical psychology literature, *reflective functioning* has been used to describe a persons ability to reflect on experiences, draw inferences about behavior from these reflections, and then use those inferences to construct and develop representations of the self (Katznelson, 2014). Research which has investigated reflective functioning has demonstrated that changes in reflective functioning are linked to self-concept change. For example, in research with persons affected by borderline personality disorder, (a condition which is characterized by an unstable sense of self) Levy et al. (2006) found that improvements in reflective functioning were associated with improvements in self-representations and a more integrated sense of self.

Another reason for thinking that self-reflection should represent an important mechanism in self-concept construction and development comes from research which has utilized the self-referential memory paradigm. In a typical self-referential memory paradigm study, different word categories (i.e., traits and adjectives verse semantically and orthographically related words) are presented to participants who are instructed to remember them at exposure and then asked to recall them at a later time (Rogers et al., 1977). The self-reference effect describes the tendency for participants to retrieve traits and adjectives that are self-related more successfully than words that are semantically or orthographically related (Symons and Johnson, 1997). Schizophrenia is another condition of which an unstable sense of self represents a core feature (see Sass and Parnas, 2003), and research has demonstrated that persons affected by schizophrenia tend to display weaker self-reference effects compared to healthy controls which researchers have interpreted as an indication of reduced self-reflective capacity (Harvey et al., 2011).

There are also several reasons for thinking that meaning-making tendencies should play an important role in self-concept construction and development in addition to the emphasis placed upon this process by narrative identity theorists as noted previously. Firstly, in a theoretical sense, influential thinkers such as Erikson (1963), Frankl (1969), and Bruner (1990), have all argued strongly for the idea that meaning is likely to play an important role in self and identity development. At the same time, research from within the organizational psychology literature has demonstrated empirically that perceptions of meaningfulness are associated with a range of self-related outcomes. *Psychological empowerment* captures an employees cognitive-motivational stance toward their work and is comprised of four dimensions, *impact*, *competence*, *autonomy*, and of particular pertinence given the current investigation, *meaning* which reflects the degree to which one perceives their work as being personally meaningful (Spreitzer, 1995; Holdsworth and Cartwright, 2003). The importance of perceptions of meaningfulness within the context of psychological empowerment is further highlighted by Spreitzer et al. (1997, pp. 681) who argue that the dimension of meaning “serves as the ‘engine’ of empowerment.” Research exploring psychological empowerment at an individual factor level has noted that differences in meaning are positively associated with several self-related outcomes such as self-esteem and self-efficacy (McAllister, 2016).

**FIGURE 1:**
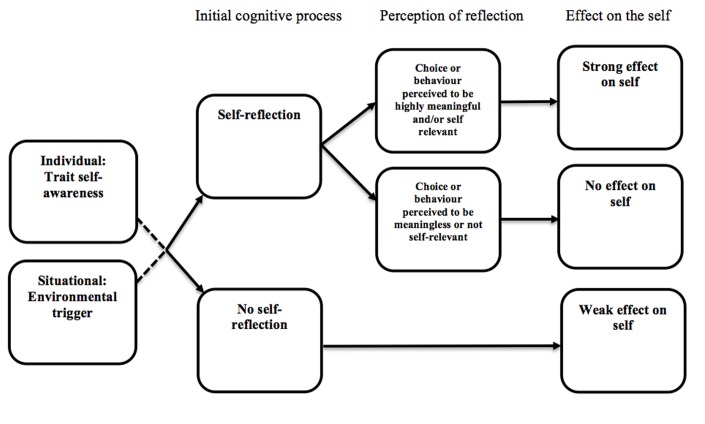
Self-reflection model.

In our own research we have found that individual differences in trait self-awareness are associated with perceptions of choice meaningfulness within a decision-making context (Dishon et al., under review). Based on pre-existing literature which has explored self-awareness more generally (e.g., Morin, 2011) we defined trait self-awareness as individual differences in the capacity to access knowledge, insight and understanding of internal self-related experiences. We found that participants with higher levels of trait self-awareness perceived significantly more meaning in a series of minor experimentally induced choices compared to those with lower levels of trait self-awareness. Moreover, this difference remained irrespective of whether or not participants were told that their choices were diagnostic of important personal characteristics. We concluded from this research that individuals high in trait self-awareness are more likely to reflect on their choices and more likely to find them meaningful than individuals low in trait self-awareness. Extending on this work and drawing upon the literature previously presented, in the present paper we propose and explore a theoretical model (see **Figure 1**) that articulates how self-reflection and perceptions of meaningfulness might affect the self within a choice context.

### Overview of the Self-Reflection Model

The assumptions underpinning this model are that when one is presented with a potential trigger event such as (but not limited to) a choice or behavior, the self will be affected (i.e., the choice/behavior will inform the self) as a consequence of (a) whether or not self-reflection takes place, and (b) the degree to which the choice is perceived to be personally meaningful. Moreover, (c) whether or not reflection takes place may be determined by individual or situational factors. For example, individuals with higher levels of trait self-awareness may be more predisposed to engage in self-reflective reasoning, whereas for others, situational cues such as an unexpected occurrence or a prompt from a third party might act as the catalyst for self-reflective reasoning. Several predictions arise from the model.

Prediction 1: If self-reflective reasoning does occur and the choice or behavior is perceived to be highly meaningful, then self-perception will occur (by which we mean the self-concept will be modified or changed as result of the behavior or action).

Prediction 2: If self-reflective reasoning does occur and the level of personal meaning associated with the choice or behavior is perceived to be low, its affect on the self will be weak or absent.

Prediction 3: If no self-reflective reasoning occurs there will be a weak effect on the self through an automatic self-perception process. Rather than predict no effect on the self in the absence of self-reflection, we allow for the possibility of an automatic or implicit self-perception process to occur because research has demonstrated that the self-concept can be impacted even in the absence of explicit reasoning. For example, in one demonstration of this type of effect, Klimmt et al. (2010) observed that exposing participants to different types of characters in video games led to automatic shifts in self-perception as measured in a follow up Implicit Association Test.

Prediction 4: Individuals high in trait self-awareness will be more likely to engage in self-reflective reasoning than individuals low in trait self-awareness^1^.

Prediction 5: Individuals low in trait self-awareness will engage in self-reflective reasoning only if prompted, or if some other situational cue triggers self-reflection.

Although narrative identity researchers have primarily looked at self-reflective reasoning in the context of autobiographical memories (see, Pasupathi, 2015), in the present study we sought to initially test the veracity of our self-reflection model on a smaller scale in a relatively minimal decision-making context. We did so for several reasons. First, decision-making lends itself well to experimental testing (Carroll and Johnson, 1990). This is important because as noted earlier, to date, research investigating the relationship between self-reflective reasoning and the self has largely been correlational by design and attempts to test this possibility experimentally have been insufficient (Adler et al., 2016). Second, consumer decision-making research has suggested that self-narratives often arise in every day decision-making contexts (Phillips et al., 1995) and some narrative identity scholars have argued that day-to-day narratives which might not be overtly autobiographical nevertheless remain tightly linked to self and identity (Bamberg, 2011; Pasupathi, 2015). Third, behaviorist and cognitive theories (i.e., self-perception theory and cognitive dissonance theory) suggest that the self is often informed by after-the-fact explanations for behaviors or *post hoc* reasoning for choices (Brehm, 1956; Festinger, 1957; Bem, 1972). Another reason for thinking that self-reflection could impact self-perception stems from research by Wilson et al. (1993) which demonstrated that self-reflection can impact attitudes and post-choice satisfaction within a decision-making context.

### Identity from a Social Identity Framework

From the view of social identity theory, our sense of identity is heavily influenced by the social groups that we belong to (Tajfel and Turner, 1986). Social identity as originally conceptualized by Tajfel (1981) refers to “…that part of an individual’s self-concept which derives from his knowledge of his membership of a social group” (p. 255). According to the theory, we come to identify with certain social groups based upon the extent to which we think we share similarities with other group members. Then, in order to maintain a positive sense of our social identity we try to ensure that our group (the in-group) is favored over other out-groups. One way of doing this is by favoring one’s in-group and discriminating against the out-group. Within the social identity literature, the extent to which we feel similar to, like, or favor other in-group members is indicative of the extent to which our identification with that group has been incorporated into our self-concept (Hogg, 1992, 1993; Ellemers et al., 1999; Leach et al., 2008). The minimal group paradigm which facilitates the measurement of in-group favoritism and out-group discrimination is one way of measuring the extent to which group membership has been incorporated into the self-concept and therefore had an effect on social identity (Otten, 2016).

In a typical minimal group paradigm experiment, participants are randomly allocated to a group and then asked to concurrently distribute resources to in-group and out-group members on allocation matrices specifically designed to measure allocation strategies that favor the in-group and∖or discriminate against an out-group (Tajfel et al., 1971). Research in the field has consistently demonstrated that even when people are led to believe that their assignment to a group is for a trivial reason, such as their preferences for abstract artwork, they still tend to allocate resources more favorably to in-group members (Otten, 2016). Whilst researchers have often been interested in using this methodology to investigate topics such as prejudice and discrimination, the allocation of resources within a minimal group paradigm environment need not be used exclusively for this end (Bourhis et al., 1994). The allocation of resources within a minimal group paradigm context can also serve as a subtle and discreet measure of the degree to which group membership has been incorporated into the self-concept and one’s sense of social identity more generally (Otten, 2016). Another way that social identity researchers have measured the extent to which commitment to a group can impact one’s self-concept and sense of identity is by measuring self-reported liking of, and similarity with, other anonymous in-group members (e.g., Hogg, 1992, 1993; Ellemers et al., 1999; Leach et al., 2008). Ellemers et al. (1999) research is also important in the context of the current study because it demonstrates that social identification is more strongly affected when people are able to self-select into a group (as opposed to being assigned a group) and it would seem reasonable to think that self-reflective reasoning is a process that could be quite important for self-selection decisions.

### The Current Study

In recent research in our lab we investigated the connection between self-reflective reasoning within a decision-making context and the self. We found that the degree of personal meaning that was given to a trivial choice was associated with individual differences in trait self-awareness (Dishon et al., under review). In the present study we sought to extend this research by investigating further if the cognitive process of engaging in self-reflective reasoning could affect one’s sense of identity. We also sought to explore whether an effect of this kind might be impacted by the extent to which one felt as though their reasoning had been personally meaningful and also moderated by individual differences in trait self-awareness. To test this model we developed an experiment that utilized and extended upon traditional minimal group paradigm work. Participants were randomly assigned to either an experimental or control condition. In the experimental condition participants were prompted to engage in self-reflective reasoning immediately after making painting choices whereas in the control condition participants went on to allocate resources immediately after selecting paintings. We used in-group∖out-group allocation strategies as one dependent measure of identity and we also used similarity and liking ratings with in-group∖out-group members as additional dependent measures of identity.

Based on the proposed model we hypothesized that participants who are relatively high in trait self-awareness would be more likely to spontaneously self-reflect on their choices and therefore be relatively unaffected by the self-reflection prompt manipulation. As such it was expected that for these participants, self-perception would be related to the perceived meaningfulness of their painting choices more so than condition. We also expected that participants who are relatively low in trait self-awareness would be less likely to spontaneously self-reflect on their choices and therefore more greatly affected by the self-reflection prompt manipulation. As such it was expected that for these participants, self-perception would be related to the perceived meaningfulness of their painting choices only in the experimental condition (i.e., when they have been prompted to self-reflect.)

## Materials and Methods

### Participants

Two hundred and six undergraduate psychology students voluntarily participated in the study in exchange for course credit. During the procedure, a manipulation check was administered to ensure that participants had attended to feedback regarding group allocation (the details of which are explained further in the Procedure section below). The responses of 32 participants who failed the manipulation check were discarded leaving a remaining pool of 174 participants (139 female, 35 male) with a mean age of 33.06 years (*SD* = 11.78). The difference in failure rates between conditions was not significant (*p* = 0.518). Ethical approval for the study was provided by Swinburne University’s Human Research Ethics Committee (SUHREC).

### Materials

#### Identity

Effects of the experimental manipulation on identity were inferred by, (a) the extent to which participants incorporated their in-group identification into their self-concept and measured by participant’s in-group favoritism when distributing resources to in-group∖out-group members on Tajfel matrices and, (b) participant’s self-identification with in-group∖out-group members which was assessed by measuring their liking of, and perceived similarity with, in-group∖out-group members.

##### Tajfel matrices

Tajfel matrices consist of six matrices in which participants are asked to allocate resources concurrently to an in-group member and out-group member along a spectrum of pre-determined in-group to out-group ratios. The six matrices comprise three pairs (one of each pair is a reversed version of the original).

There are four main allocation strategies that can be measured with Tajfel matrices. *Parity* is an allocation strategy whereby the participant distributes an equal amount of resources to both in-group and out-group recipients. *Maximum In-Group Profit* is an allocation strategy that sees the greatest possible amount of resources awarded to the in-group recipient irrespective of what is awarded to the out-group recipient. *Maximum Difference* reflects a strategy that optimizes the differential allocation of resources between recipients in favor of the in-group recipient at the expense, however, of absolute in-group profit. *Maximum Joint Profit* reflects a strategy in which overall allocation of resources is maximized across both in-group and out-group.

The matrices facilitated the calculation of *pull scores* which reflected participants’ gravitation toward particular allocation strategies. Matrix pair A compared the pull of Maximum In-Group Profit and Maximum Difference (i.e., in-group favoritism) against Maximum Joint Profit. Matrix pair B compared the pull of Maximum Difference against Maximum In-Group Profit and Maximum Joint Profit. Matrix C compared the pull of Parity against Maximum In-Group Profit and Maximum Difference [See Bourhis et al. (1994) for a comprehensive and in-depth account of the procedure involved in Tajfel matrix preparation, administration, and calculation].

Following a similar procedure to Grieve and Hogg (1999) we then conducted a factor analysis of the pull scores using principal axis factoring with promax rotation to examine the possibility of computing an overall in-group favoritism score. This revealed a single in-group favoritism factor which explained 48.9% of the variance (all loadings ≥ 0.63). The items were then summed and averaged to produced an overall measure of in-group favoritism with higher scores representing greater in-group favoritism (Cronbach’s α = 0.74).

##### In-group self-identification

As other researchers have done previously (e.g., Hains et al., 1997; Grieve and Hogg, 1999), participants’ liking of, and perceived similarity with, in-group∖out-group members were recorded to measure their level of self-identification with their in-group. To do so, after being presented with pairs of de-identified paintings by Paul Klee and Wassily Kandinsky and receiving feedback that their choices indicated a preference for the work of Klee irrespective of their actual choices, (see the Procedure section below for a more detailed account of the process involved,) participants were asked to imagine themselves meeting two people, one who had a preference for Klee and the other who had a preference for Kandinsky. Participants then rated on a seven-point scale which of these two people they thought they were most similar to in general (Q1), in artistic preferences (Q2), in painting preferences (Q3), in academic ability (Q4), and in political opinions (Q5). Using the same scenario, participants were also asked to rate who they thought they would like more (Q6), who they thought they would get along with more (Q7), and who they would like to meet more (Q8). Responses on questions 1–5 were summed and averaged to calculate an overall *similarity score* with higher scores representing a greater level of similarity with an in-group member (Cronbach’s α = 0.75). Response for questions 6–8 were summed and averaged to calculate an overall *liking score* with higher scores representing a greater level of liking for an in-group member (Cronbach’s α = 0.82).

#### Meaningfulness

Meaningfulness associated with self-reflective reasoning was measured by providing participants with a five-item Subjective Meaningfulness Scale which included items such as *“I feel as though my choices were genuine”* and, *“My choices were meaningless.”* Participants were asked to indicate their level of agreement with each statement on a 5-point scale (1 = strongly disagree, 5 = strongly agree). Responses were coded so that higher scores indicated greater level of meaningfulness. A factor analysis using principal axis factoring and promax rotation revealed that all five items loaded on a single factor which explained 32.6% of the variance (all loadings ≥ 0.43). Scores were then summed and averaged and an overall meaningfulness score was calculated (Cronbach’s α = 0.69; Guttman’s Lambda 2 = 0.70).

#### Trait Self-Awareness

Trait Self-Awareness was operationalized as function of participants’ scores on the Sense of Self Scale (SOSS; Flury and Ickes, 2007) which is a single factor 12-item measure designed to assess sense of self and self-understanding (Cronbach’s α = 0.86) and the Self-Reflection and Insight Scale (SRIS; Grant et al., 2002) which is a two factor 20-item measure of self-reflection and insight (Cronbach’s α = 0.88). In the present sample, using principal axis factoring and promax rotation, both measures retained their original factor structures with the SOSS exhibiting a single factor which accounted for 36.3% of the variance and the SRIS exhibiting two factors which accounted for a combined 53.6% of the variance (Factor 1 = 34.3%, Factor 2 = 19.3%). Both measures were scored so that higher scores indicated stronger sense of self and greater levels of self-reflection and insight and both measures were significantly correlated (*r* = 0.35, *p* < 0.001). Scores on these scales were then summed to create an overall trait self-awareness score with higher scores representing greater levels of trait self-awareness (Cronbach’s α across the total 32-items = 0.77; principal axis factoring with promax rotation revealed three factors accounting for 50.6% of the variance [Factor 1 = 24.6%, Factor 2 = 21.8%, Factor 3 = 3.9%]).

#### Stimuli

Six pairs of images of paintings by Paul Klee and Wassily Kandinsky were utilized as the painting stimuli.

### Procedure

The experiment was administered online. Once consent to participate was provided, participants were informed they would be required to choose their preferred painting from six pairs of paintings which were then presented sequentially. All paintings were presented without the artists’ names attached to any of the works. After making their painting selections participants were randomly assigned to one of two conditions (a reasoning *pre* resource allocation, similarity and liking ratings condition or, a reasoning *post* resource allocation, similarity and liking ratings condition). Participants in both conditions were presented with all the same stimuli and experiences except the order of exposure was manipulated slightly between conditions as outlined below.

In the reasoning *pre* condition, after the initial painting selection phase, participants took part in the self-reflective reasoning phase. In the self-reflective reasoning phase participants were presented with and asked to reflect on a 15-item list of potential reasons for their painting selections and then presented with an open text box and asked to reflect further in their own words about their reasons for their painting choices. Following this participants were presented with and completed the subjective meaningfulness measure. Then although they remained unware to it at the time, irrespective of their actual choices participants were informed that their choices indicated that they preferred the works of Paul Klee^2^. Participants were then presented with instructions pertaining to the completion of the Tajfel matrices before moving on to complete them. Following this, participants were presented with the in-group∖out-group similarity and liking measure. Participants then completed the trait self-awareness measures before recording their gender (female, male, or other) and age. A manipulation check was then conducted whereby participants were asked to indicate who they had previously been informed that their painting choices indicated they preferred the works of (possible response were, Paul Klee, Wassily Kandinsky, or Don’t remember). Participants were then presented with a debriefing statement, informed the experiment was over and thanked for their participation.

In the reasoning *post* condition, the order of exposure was manipulated so that after making painting selections, participants were told their choices indicated a preference for Paul Klee^3^ and were administered with the matrices and in-group∖out-group similarity and liking measures *before* the self-reflective reasoning phase. After completing the choice reasoning phase and the subjective meaningfulness measure, participants in this condition were also then presented with the same trait self-awareness^4^ measures, demographic questions, manipulation check and debriefing as their counterparts in the alternate condition.

## Results

### Outlier Analysis

Three multivariate outliers (1 in the control and 2 in the self-reflection condition) were detected and removed from the analysis thereby leaving a total sample of 171 (86 in the control condition and 85 in the self-reflection condition).

### Descriptive Statistics

Descriptive statistics for trait self-awareness, choice meaningfulness, in-group similarity, in-group liking, and in-group favoritism as a function of self-reflection condition are presented in **Table 1**.

**Table 1:** Means and standard deviations for trait self-awareness, choice meaningfulness, similarity, liking, and in-group favoritism by self-reflection condition.

	Condition			
	Control group		Self-reflection group	
Measure	Mean	*SD*	Mean	*SD*
Trait self-awareness	7.30	0.93	7.49	1.04
Choice meaningfulness	4.00	0.51	3.93	0.56
Similarity	5.13	0.68	4.95	0.80
Liking	4.60	0.92	4.55	1.11
In-group favoritism	0.95	3.11	1.13	2.65

#### Effect of Experimental Manipulation on IV’s

We conducted between groups analyses to investigate if the self-reflection and control groups differed on the IV’s of choice meaningfulness and trait self-awareness as a function of the self-reflection manipulation. Independent samples *t*-test’s revealed that there was no significant difference in choice meaningfulness (*p* = 0.365) or trait self-awareness (*p* = 0.218) between conditions thereby demonstrating the IV’s were robust to the self-reflection manipulation.

### Multiple-Sample Path Analysis

We ran a multiple-sample path analysis using the structural equation modeling program MPLUS (v 7.4) to investigate if the main effects of meaningfulness and trait self-awareness as well as the interaction effects (i.e., choice meaningfulness × trait self-awareness) on the DV’s differed between the experimental self-reflection and control non-self-reflection conditions. The model tested three exogenous/independent variables all predicting the three endogenous/dependent variables, in-group favoritism, similarity and liking. The exogenous variables were the main effects of trait self-awareness and choice meaningfulness, and a trait self-awareness × choice meaningfulness interaction.

Because the parameter to case ratio was under the required minimum of 1 parameter to 5 cases (1:4.75 or 36:171) as suggested by Kline (2011), we discreetly tested each section of the model. In other words, three independent models with each of the three endogenous dependant variables were examined separately thereby ensuring that the parameter to case ratio was sufficient (i.e., 1:17.1 or 10:171). In all models the Satorra–Bentler robust estimator was used to account for multivariate non-normality, and all parameters were free across the self-reflection and control conditions. Chi-square Wald tests were utilized on a fully unconstrained model to test significant differences in the effects across conditions given the expectation that there would be differences in regression weights across groups (Muthén and Muthén, 1998–2017). There were no significant differences in the results of these separate models and the full model^5^. Given this, the parameters for the full model are presented in **Table 2**. Because the model was saturated with zero degrees of freedom fit indices are not reported.

**Table 2:** Unstandardized regression weights and Wald Tests for the multi-sample path analysis.

		Control group		Self-reflection group			
Endogenous variable	Effect	RW	*SE*	RW	*SE*	Wald	*SE*
In-group favoritism	Trait self-awareness	-0.32	0.31	0.23	0.24	0.55	0.39
	Choice meaningfulness	0.74	0.62	0.09	0.49	-0.65	0.79
	Trait self-awareness × Choice meaningfulness	0.40	0.68	-0.41	0.41	-0.81	0.80
Liking	Trait self-awareness	0.02	0.13	-0.21*	0.10	-0.23	0.16
	Choice meaningfulness	0.20	0.20	0.93***	0.21	0.73*	0.29
	Trait self-awareness × Choice meaningfulness	0.20	0.22	-0.65***	0.16	-0.85**	0.27
Similarity	Trait self-awareness	0.06	0.08	0.04	0.08	-0.02	0.11
	Choice meaningfulness	0.32*	0.14	0.59***	0.14	0.27	0.20
	Trait self-awareness × Choice meaningfulness	-0.05	0.13	-0.33*	0.10	-0.28^†^	0.17

*RW, regression weight; SE, standard error. ^†^*p* < 0.10. ^∗^*p* < 0.05, ^∗∗^*p* < 0.01, ^∗∗∗^*p* < 0.001*.

### Main Effects of Choice Meaningfulness and Trait Self-Awareness

The results in **Table 2** reveal that there was a significant main effect for choice meaningfulness in both the control and self-reflection conditions for similarity, however, Wald tests reveal that the difference in effects between conditions was not significant. This suggests that higher choice meaningfulness scores were associated with higher similarity scores in both the self-reflection and control conditions. The results in **Table 2** also demonstrate that there was a significant main effect of choice meaningfulness for liking in the self-reflection condition whereas the main effect of choice meaningfulness for liking in the control condition was not significant. The Wald test demonstrates that this difference in effects between conditions was significant, suggesting that higher choice meaningfulness scores were associated with higher liking scores in the self-reflection condition only. Whilst there was also a significant main effect of trait self-awareness on liking in the self-reflection condition, the Wald test demonstrates that this was not significantly different from the non-significant main effect of trait self-awareness in the control condition.

### Trait Self-Awareness × Choice Meaningfulness Interaction Effects

**FIGURE 2:**
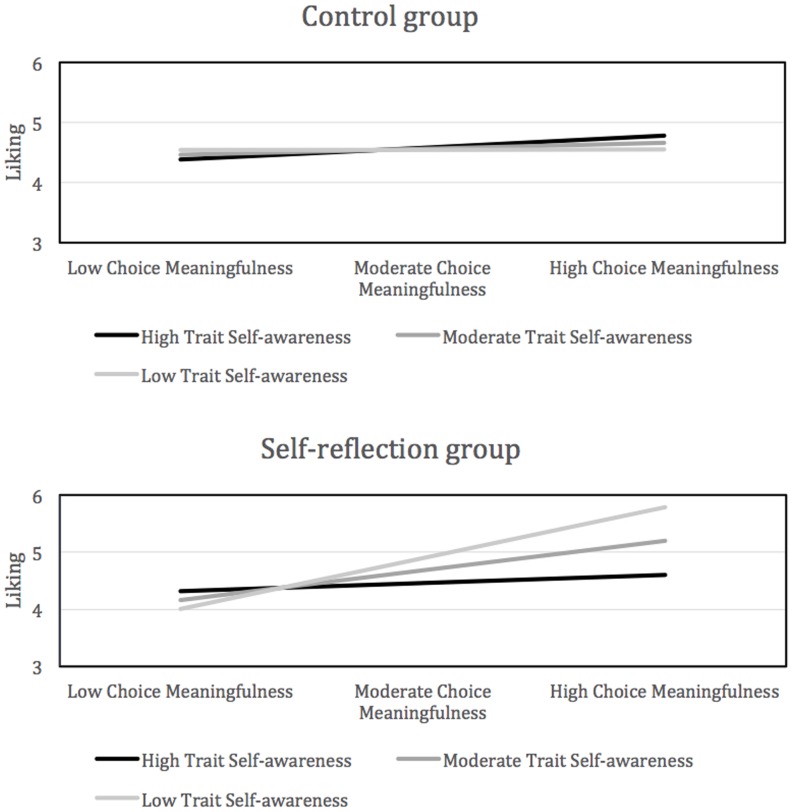
Predicted liking scores by trait self-awareness and choice meaningfulness groups across conditions. Groups were defined as 1 standard deviation above (high) and below (low) the mean (moderate).

As seen in **Table 2**, for liking, the interaction between trait self-awareness and choice meaningfulness was only significant in the self-reflection condition and as the significant Wald test demonstrates, the strength of this interaction effect was also significantly different between the control and self-reflection conditions (see **Figure 2**).

The results in **Figure 2** suggest that for the self-reflection group the relationship between choice meaningfulness and liking strengthens as trait self-awareness scores decrease. That is, higher choice meaningfulness scores appear to be strongly associated with higher liking scores for those with lower trait self-awareness scores. This demonstrates a stronger impact of the self-reflection manipulation on participants lower in trait self-awareness and a reduction in the impact of the manipulation as trait self-awareness levels increase. Parallel trends were observed for similarity though the Wald test was only marginally significant.

## Discussion

The present study explored whether engaging in self-reflective reasoning could affect in-group identification and thereby demonstrate an effect of self-reflection on indicators of social identity and the self-concept. The possibility that such an effect could be impacted by the perceived level of meaningfulness associated with reasoning, and modulated by individual differences in trait self-awareness was also explored. Based on previous research, we developed a model which predicted that participants with higher levels of trait self-awareness would be minimally affected by the self-reflection manipulation. It was therefore hypothesized that for these participants self-perception would be related to the perceived meaningfulness of their painting choices more so than condition. The model further predicted that the self-reflection manipulation would have a greater impact on participants lower in trait self-awareness. Consequently, it was further anticipated that for these participants, self-perception would be related to perceived meaningfulness of their painting choices only in the experimental condition (i.e., when they were prompted to self-reflect). Participants’ in-group similarity and liking ratings (but not in-group favoritism allocations) supported these predictions and provided general support for the theoretical model proposed earlier.

Considering first the main effects of meaningfulness across conditions, the data demonstrated that whilst greater levels of meaning were associated with greater in-group similarity scores in both conditions, greater levels of meaning were only associated with in-group liking scores in the self-reflection condition. Taken together these results suggest that compared to the control condition, in the self-reflection condition stronger perceptions of meaningfulness led to stronger in-group identification. This is in line with predictions 1 and 2 relating to the self-reflection pathway in the theoretical model. Additionally, in line with prediction 3 of the theoretical model relating to the no self-reflection pathway, the effect of meaning on in-group perceptions in the control condition was smaller relative to the self-reflection condition. Moreover, the fact that there was no interaction between trait self-awareness and choice meaningfulness in the control group is suggestive of the possibility that in this condition, the main effect of meaning on in-group liking was the product of automatic or implicit processing because it occurred in the absence of any situational prompting and was not also impacted by pre-existing individual dispositions toward spontaneous self-reflection.

At the same time, the interaction between trait self-awareness and choice meaningfulness in the experimental condition indicates that the relationship between perceptions of choice meaningfulness and in-group liking strengthens as trait self-awareness levels decrease. This result suggests that the situational self-reflection prompt exhibited a stronger impact on participants who were less inclined (in terms of individual disposition) to engage in spontaneous self-reflection, and had less of an impact on participants with greater levels of individual disposition toward spontaneous self-reflection. This provides support for the hypothesis presented earlier and is also in line with predictions 4 and 5 of the theoretical model. A similar trend was also noted for in-group similarity, however, the difference in the strength of the effect between conditions was only marginally significant.

### Limitations and Future Directions

One aspect of the study that could be viewed as both a limitation and strength is the way in which identity was measured. In the present study utilizing an already established experimental paradigm we developed a subtle way of testing the effect of self-reflective reasoning on identity. However, the cognitive process we were investigating was a process theorized from a narrative identity theory perspective, and the methodology used was born out of the social identity theory literature. On the one hand this approach represented a strength of the design in that it facilitated a discreet measurement of the effect of self-reflective reasoning on identity. At the same time, however, there are differences in the way that identity is conceptualized across both projects. To provide a stronger test of the hypothesis that self-reflective reasoning can affect narrative identity, experimental work with a more traditional dependent measure of narrative identity would be useful.

Another limitation was the way in which trait self-awareness was operationalized. In the present study trait self-awareness was operationalized as a function of participants’ scores on the SOSS and the SRIS. Whilst we had good reason to combine and operationalize these measures as a means of measuring trait self-awareness, future research aimed at the development of a dedicated measure of trait self-awareness would be worthwhile.

Though we were able to ensure that the modeling we conducted was sufficiently powered the present study could have benefited from a larger sample size. In the present study the parameter to cases ratio for the overall model was less then recommended (e.g., Kline, 2011). Therefore as outlined in the results section, to ensure that our modeling was sufficiently powered we initially computed three discreet models, one for each dependent variable. Although there was no significant difference in the outcomes between the individual and the combined models, in future to avoid the necessity of running independent models for each dependent variable it would be beneficial to recruit a larger sample which meets the parameter to cases ratio for the entire model in the first instance.

It is also possible that the deception that we engaged in (i.e., providing all participants with feedback that they preferred the work of Klee, irrespective of their actual choices) could have raised suspicions amongst participants who may have actually had some pre-existing knowledge of Klee and∖or Kandinsky (i.e., the artists whose works were used as the choice stimuli). Whilst we did include a manipulation check to ensure that the deception had had its intended effect, in future research, to address this issue more comprehensively it would be beneficial if participants were also directly questioned about their pre-existing knowledge of the artists whose works are used as the choice stimuli. Another way that this issue could be controlled for in the future would be to use the works of unknown artists as the choice stimuli.

### Implications

The results of the present study may be of value to researchers who are interested in the developmental trajectory of narrative identity and autobiographical reasoning. Previous research looking at the development of narrative identity has suggested that the ability to cultivate a life-story tends to arise on average by about 14 years of age and that this is preceded by autobiographical reasoning for memorable life events which tends to first arise between the ages of nine and ten (Bohn and Berntsen, 2008). Little is known, however, about the antecedents to the onset of autobiographical reasoning processes. Whilst it could be the case that development of autobiographical reasoning processes occurs in a stepwise fashion with little preceding them, the results of this study which demonstrate that reasoning about a trivial choice can effect the self and identity, beg the question that perhaps autobiographical reasoning processes develop as a continuous extension of more basic self-reflective reasoning processes which develop earlier in childhood. Perhaps it is the practice of more basic self-reflective reasoning which lays the cognitive foundations for, and facilitates the development of, more advanced autobiographical reasoning. One piece of recent research which dovetails with this idea comes from Bryan et al. (2014) who found in their work that children between the ages of three and six are already engaging in everyday decision making behaviors that are motivated by their developing sense of self and identity.

The fact that we observed significant effects for in-group identification and no effects on in-group favoritism has implications for researchers interested in intergroup discrimination and self-categorization. Specifically, the effects that we observed for in-group liking and in-group similarity suggest that self-categorization is likely to be influenced by both self-reflection and the level of subjective meaningfulness associated with choices or behaviors on which self-categorization is based. The absence of any effect on in-group favoritism suggests that intergroup discrimination is unlikely to be substantially impacted by self-reflection or choice meaningfulness. Research which has investigated positive-negative asymmetry within a minimal group paradigm context may help explain the discrepancy in effects between the attitudinal and behavioral measures. Positive-negative asymmetry research (see, Buhl, 1999; Mummendey et al., 2000) has demonstrated that group members tend to display stronger in-group preferences on positive stimuli compared to negative stimuli (i.e., evaluations of well regarded attributes such as creativity or intelligence, verses allocations of aversive noise). Given this research, one possibility that exists then is that in the current study, the attitudinal in-group liking and similarity measures which required participants to evaluate group members along positive dimensions were perceived more favorably compared to the behavioral measure of in-group favoritism which required participants to make allocation choices that had the potential to disadvantage out-group members.

Another possible explanation, however, for the absence of an effect on in-group favoritism could be due to aspects of the wider cultural climate within which participants were located at the time. Specifically, when this experiment took place Australia remained in the midst of a nation-wide debate regarding the legalization of same-sex marriage. Within the context of this debate university students have had strong messages of social justice and fairness directed at them at a cultural level. For example, the National Union of Students, which is the nations peak student representative body strongly advocated for students to support marriage equality (Barlow, 2017). Given the cultural climate and the strong messages of social justice and fairness directed at students during the period in which this experiment took place, it is possible that in the allocation matrix tasks participants felt more compelled to engage in resource allocations which emphasized parity rather than discrimination. At the same time, in-group similarity and in-group liking ratings may have remained relatively immune to the impact of these cultural messages because perceptions of in-group identification do not necessarily equate to out-group discrimination and therefore do not have the same kinds of implications for one’s sense of fairness or social justice.

The present study may also have some implications for researchers whose work is informed by self-perception and cognitive dissonance theories. The results of the present study suggest that the application of self-reflection theory could be useful in some contexts in which cognitive dissonance and self-perception theories are not well positioned to explain the effect of choice or behavior on the self. According to self-perception theory (Bem, 1972), after-the-fact explanations for behavior are generally limited to attributions about the internal (dispositional) or external (situational) cause of a behavior and are also only likely to occur in circumstances in which there is a weak or non-existent pre-existing explanation for the behavior. From the view of cognitive dissonance theory (Festinger, 1957), *post hoc* reasoning about choices is limited to choices that induce dissonance and are motivated by a desire to reduce dissonance. Our model, however, suggests that choice or behavior is likely to effect the self as a consequence of whether it was actually perceived to be personally meaningful and that this needn’t be exclusive to dissonance inducing choices, nor to behaviors for which one does not have a pre-existing explanation.

## Conclusion

Within the narrative identity literature, reflecting on life events in a personally meaningful way has been conceptualized as one of the key psychological mechanisms underpinning our sense of identity. To date, however, research on this issue has been largely correlational with little causal evidence available to confirm or disconfirm this claim. In the present study we sought to test experimentally if this cognitive process theorized to be so vital for identity development, could have a causal effect on self and identity. We also sought to explore the possibility that such an effect could be impacted by the level of meaningfulness associated with self-reflective reasoning, and modulated by individual differences in trait self-awareness. The results of this study largely supported our hypothesis and the proposed model from which those predictions were derived. For participants who were high in trait self-awareness, being prompted to engage in self-reflective reasoning mattered little. For this group of participants, in-group liking and similarity was related to perceptions of subjective meaningfulness relatively equally across conditions. At the same time, however, for participants low in trait self-awareness, being prompted to engage in self-reflection mattered a great deal. For these participants, subjective meaningfulness moderated in-group liking and similarity only when they had been prompted to engage in self-reflection. Overall the results of this study provide evidence to suggest that engaging in self-reflective reasoning can affect the self and identity and that this effect is impacted by both choices meaningfulness and individual differences in trait self-awareness.

## Author Contributions

ND, JAO, CC, and JK all contributed to the paper and approved it for publication.

## Conflict of Interest Statement

The authors declare that the research was conducted in the absence of any commercial or financial relationships that could be construed as a potential conflict of interest.

## Footnotes

1 Even though we predict that those high in trait self-awareness will be more likely to engage in self-reflection, given the magnitude of the relationship between trait self-awareness and perceptions of choice meaningfulness noted in our previous study (Dishon et al., under review) there is still scope for those high in trait self-awareness to find their choices meaningless.

2 Participants remained unware of this deception until they were debriefed at the end of the study.

3 As was the case in the reasoning pre condition, participants in this condition also remained unaware to the fact that they had received this feedback irrespective of their actual choices up until they were debriefed at the end of the study.

4 Even though individual differences in trait self-awareness represent a starting point in the theoretical model, in both conditions the trait self-awareness measure was administered after the self-reflection manipulation and in-group∖out-group ratings had taken place because we wanted to avoid potentially priming self-reflection processes in participants prior to their exposure to the self-reflection manipulation.

5 We also unpacked the in-group favoritism factor and ran the model on each of the individual pull scores. There was no significant difference between these models and the models using the in-group favoritism factor.
